# Overview of Viral Pneumonia Associated With Influenza Virus, Respiratory Syncytial Virus, and Coronavirus, and Therapeutics Based on Natural Products of Medicinal Plants

**DOI:** 10.3389/fphar.2021.630834

**Published:** 2021-06-21

**Authors:** Ziwei Hu, Jinhong Lin, Jintao Chen, Tengxi Cai, Lixin Xia, Ying Liu, Xun Song, Zhendan He

**Affiliations:** ^1^ School of Basic Medicine, School of Pharmaceutical Sciences, Health Science Center, Shenzhen University, Shenzhen, China; ^2^ College of Pharmacy, Shenzhen Technology University, Shenzhen, China

**Keywords:** viral pneumonia, medicinal plants, natural compounds, influenza virus, respiratory syncytial virus, coronavirus

## Abstract

Viral pneumonia has been a serious threat to global health, especially now we have dramatic challenges such as the COVID-19 pandemic. Approximately six million cases of community-acquired pneumonia occur every year, and over 20% of which need hospital admission. Influenza virus, respiratory virus, and coronavirus are the noteworthy causative agents to be investigated based on recent clinical research. Currently, anaphylactic reaction and inflammation induced by antiviral immunity can be incriminated as causative factors for clinicopathological symptoms of viral pneumonia. In this article, we illustrate the structure and related infection mechanisms of these viruses and the current status of antiviral therapies. Owing to a set of antiviral regiments with unsatisfactory clinical effects resulting from side effects, genetic mutation, and growing incidence of resistance, much attention has been paid on medicinal plants as a natural source of antiviral agents. Previous research mainly referred to herbal medicines and plant extracts with curative effects on viral infection models of influenza virus, respiratory virus, and coronavirus. This review summarizes the results of antiviral activities of various medicinal plants and their isolated substances, exclusively focusing on natural products for the treatment of the three types of pathogens that elicit pneumonia. Furthermore, we have introduced several useful screening tools to develop antiviral lead compounds.

## Introduction

Community-acquired pneumonia (CAP) is a commonly encountered lung inflammation involving the alveoli resulting from the lower respiratory tract infection that occurs in patients without recent health care exposure. CAP is responsible for the high rate of morbidity and mortality worldwide. As much as 5.6 million cases of CAP occur annually, and more than 20% of which need hospital admission (Niederman et al., 2001). According to the World Health Organization (WHO), 15% of children under 5 years of age die from pneumonia (WHO 2020b); CAP is the eighth leading cause of death in the United States with approximately 50,000 people dying from the disease each year (CDC 2018; CDC 2019a; Heron 2019).

Recent surveys show that viruses are the major cause of CAP. A prospective study based on real time-PCR (RT-PCR) technique revealed that viral respiratory tract infections are highly prevalent among hospitalized CAP patients with immunodeficiency and low immune function (Tatarelli et al., 2019). Viral respiratory infection is common in pneumonia and is present in approximately 25% of patients with CAP. With the widespread introduction of improved diagnostic tests, at least 26 viruses associated with CAP have now been identified (Ruuskanen et al., 2011). Among viral pathogens, respiratory syncytial virus (RSV) predominantly remains the viral agent of severe CAP around the world (Ebbert and Limper, 2005; Lee et al., 2013; Mackenzie et al., 2019; Seidenberg 2019). Influenza virus (IFV) is the most common cause of viral pneumonia, following RSV, among 4,765 adults hospitalized with influenza, with 1,392 (29%) having pneumonia (Cantan et al., 2019). Recently, new pathogens discovered in patients infected with CAP, which have so far spread from China to 216 countries through rapid and frequent international air travel causing more than 0.6 million deaths worldwide, were associated with severe acute respiratory syndrome coronavirus 2 (SARS-CoV-2)–related 2019 novel coronavirus disease (COVID-19).

Currently, vaccines and antiviral agents have been developed as therapeutics to treat viral pneumonia. The two major antivirals used to treat influenza are neuraminidase (NA) inhibitors (peramivir, oseltamivir, laninamivir, and zanamivir) and M2 ion channel inhibitors (rimantadine and amantadine) and can be used to suppress the incidence of complications such as pneumonia (Moscona 2005; Gavigan and McCullers, 2019; Arabi et al., 2020). Nevertheless, M2 inhibitors are not widely used in clinical practice since only type A strain has M2 ion channel protein, While, M2 inhibitors, such as amantadine and rimantadine, doesn't work on the emergence of drug-resistance mutations in M2 proton channel (Hussain et al., 2017). A cohort study supports ribavirin therapy, which was approved by the Food and Drug Administration (FDA), as the primary treatment for infants and young children with RSV-associated pneumonia (Gomez et al., 2014). In contrast to IFV and RSV, experience with antiviral projects for coronavirus-associated CAP is scarce, with current knowledge coming mainly from case studies and surveillance data from clinical treatment.

Given that the treatment protocols of specific virus still lag behind for viral pneumonia, and short of miracle drugs, there is still a crying need for exploring new medicines to treat viral pneumonia. Natural products with antiviral efficacy, which are abundant in medicinal plants, are worth developing and utilizing as an alternative pharmacotherapy for treating CAPs. In the past,the discovery of antiviral lead compounds from various promising medicinal plants was limited because of, for one thing,a higher frequency of mutations and, for another, lack of chemical techniques for the identification of novel plant-based antiviral natural compounds. Thus far, a total of 1,073 small molecule new chemical entities (NCEs) have been approved for marketing from 1981 to 2010, among which 64% of the NCEs are identified as natural products or natural product-derived (Newman and Cragg, 2012), and it is considered that medicinal plants are still the primary sources of pharmacologically bioactive compounds for the therapeutics of viral pneumonia throughout history. In addition, challenges involved in frequent virus mutation, as well as the onset of viral resistances toward current antiviral agents, enhance an increasing interest for natural products as antiviral candidates. Nowadays, improved techniques in silico, such as high-throughput screening, molecular-docking, pharmacology network, and so on, strongly contribute to the isolation of potential drugs.

The aim of this review is to summarize the results on the antiviral activities of various isolated compounds from different kinds of plants, elucidating the latent mechanisms and potential interactions with related targets. This review will exclusively focus on natural products for the treatment of the above three important types of pathogens that elicit pneumonia, and other pathogens need not be discussed. Meanwhile, we have introduced some useful *in silico* methods to develop drugs from medicinal plants.

## Etiology and Current Antiviral Interventions

### Respiratory Syncytial Virus

Human respiratory syncytial viruses (HRSVs) are deemed as highly infectious pathogens that induce acute lower respiratory tract illness (ALRTI), infecting and rendering diseases in individuals of all ages, particularly in children under 5 years and in adults over the age of 65. Since it is difficult to distinguish pneumonia and bronchiolitis clinically from radiographically, numerous epidemiological studies now follow the WHO recommendation to regard any RSV-associated ALRTI as pneumonia (Borchers et al., 2013). A recent systematic review pointed out the global number of cases of RSV pneumonia in 2015 at 33.1 million, of which 3.2 million hospital admissions and 59,600 deaths with 45% of cases occurring before 6 months of age (Shi et al., 2017a).

HRSV is a pleomorphic, enveloped, cytoplasmic virus with single-stranded, nonsegmented, and negative-sensed RNA genomes of 15.2 kb that belong to the family of Paramyxoviridae of the order *Mononeavirales*, genus *Pneumovirus*, subfamily *Pneumovirinae* (Bonnet et al., 2005; Fodha et al., 2008). Its antigen of single serotype split into two subgroups, A and B, which are further divided into 13 and 20 genotypes, respectively (Anderson et al., 1985; Pangesti et al., 2018). Both groups can spread simultaneously during outbreaks, but the proportions of A and B, as well as subtypes, vary yearly.

Therapeutic drugs for HRSV have been designed to target three major pathways of the virus cycle based on the virus structure, such as entry, replication, and transcription. The genomic RNA is associated with four nucleocapsid/polymerase proteins: nucleoprotein N, phosphoprotein P, transcription processivity factor M2-1, and the large polymerase subunit L. Three encoded transmembrane surface glycoproteins on envelope participating in maximally efficient fusion contributed to infectivity, including the major attachment protein G, the fusion protein F, and the small hydrophobic (SH) protein, which remain the targets regarded most important for antiviral agents development.

The treatment of HRSV pneumonia is supportive. At present, two antiviral agents approved by the FDA are considered for optimal therapies to prevent and treat HRSV infection in children and infants with high risk. Ribavirin is a guanosine analog commonly administered in the form of erosolization, whereas intravenous ribavirin is not commercially available in the majority of countries. Multiple studies suggest that erosolized ribavirin mostly show its effectiveness at early stages of infection. Nevertheless, the application of erosolized ribavirin remains controversial owing to drug delivery, concerns about health risks for caregivers, as well as potential side effects (anemia, etc). Another successful agent allowed for human use known as a humanized monoclonal antibody against F glycoprotein. The clinical results of two randomized trials of prophylaxis with palivizumab provided the basis for FDA approval (Feltes et al., 2003). One carried out a 55% entire drop in hospital admission for RSV, corresponding to a relative reduction of 39% in children with CLD. Motavizumab is an investigational monoclonal antibody (mAb), another humanized IgG1 monoclonal antibody with a higher affinity compared to palivizumab, and can prevent serious diseases resulting from RSV in high-risk pediatric patients, and yet, the New Drug Application (NDA) of the MedImmune for motavizumab had been rejected by the FDA.

New and affordable therapeutic or prophylactic tools are urgent to develop due to a high economical cost of the current treatment. Furthermore, viral genetic mutations that allow for escaping bring about challenges in the development of antiviral agents. Therefore, there is an urgent necessary for patients to seek for new antiviral drugs.

### Influenza Virus

Seasonal influenza-associated severe pneumonia can lead to 6–29% of substantial mortality (Oliveira et al., 2001; Murata et al., 2007; Paules and Subbarao, 2017). Influenza infection accounts for the susceptibility to pneumonia by a factor of ∼100, while approximately 25% of pneumonia patients may exacerbate as continuum of the acute respiratory distress syndrome (ARDS) (Rello and Pop-Vicas, 2009; Shrestha et al., 2015). There are four pathways for IFV to trigger pneumonia, which are primary influenza pneumonia, secondary bacterial pneumonia, pneumonia due to unusual pathogens or in immunocompromised hosts, as well as deteriorations of chronic pulmonary diseases (Rothberg et al., 2008). During the 2009 pandemic, severe influenza pneumonia shapes the outcome of concurrent bacterial superinfection developed in 4–24% of cases caused by microorganisms, such as *Staphylococcus aureus*, *Chlamydia pneumoniae*, β-hemolytic *streptococci*, and *Legionella pneumophila* (Gerber et al., 1978; Miyashita and Matsushima, 2000; Johnson et al., 2008; Louie et al., 2009; Shrestha et al., 2015). Risk factors for progression to pneumonia were an absolute lymphocyte count less than 200 cells/ml besides that not receiving influenza-directed antiviral therapy (Chemaly et al., 2006). A large study examining children hospitalized with influenza from 2007 to 2015 noted that only 69% received antiviral treatment (Gavigan and McCullers, 2019).

IFV with a negative-sense, single-stranded, and segmented RNA genome belongs to the family Orthomyxoviridae, which are further classified into IFV A, B, C, and D. In previous studies, influenza A and B viruses whose highly contagious pandemic are primarily responsible for seasonally acute respiratory disease that outbreaks and spreads worldwide give rise to increased ICU admission, mortality, and a substantial economic burden. Compared with influenza B, researchers tend to be preferentially concentrated on type A, because it is generally considered the predominant type in influenza disease. However, a study elucidated that pneumonia is more likely to occur in men with a confirmed type B infection and presenting with shortness of breath (Dai et al., 2020).

Nowadays, there are 131 subtypes of A strains that have been identified in nature, based on hemagglutinin (H1-H18) and neuraminidase (N1-N11) transmembrane glycoproteins, among which A(H1N1) and A(H3N2) routinely circulate worldwide (CDC 2019b). Nevertheless, seasonal H1N1 strain had been replaced by the 2009 H1N1 pandemic strain (H1N1 pdm09), and H7N9 strain was discovered as a novel subtype in 2013. Additionally, influenza B virus is divided into two lineages: B/Yamagata and B/Victoria (Jha et al., 2020). Since the 2009 pandemic year, the primary circulating A strains have been the H1N1 pandemic strain and an H3N2 strain, whereas both the types of influenza B clades have cocirculated according to national surveillance reports (Gavigan and McCullers, 2019). The frequency of primary viral pneumonia differed among the virus-associated pneumonia subtypes (pH1N1, 80%; H3N2, 26.5%; and B, 31%) (Ishiguro et al., 2016).

In brief, two groups of antivirals are available for the treatment of influenza: the neuraminidase inhibitors (NAIs), and the virus polymerase inhibitors. Amantadine, which has been used to treat influenza for many decades, has been found to target the M2 ion channel that interferes with viral uncoating following entry into the host cell. In addition, amantadine can affect the pH regulation of vesicles involved in the transport of viral glycoproteins to the cell surface during assembly (Pinto et al., 1992). As significant rates of resistance to the adamantanes and to its 10-fold more active derivative, rimantadine, this medication is being phased out since 2005 (Monto and Arden, 1992). The hemagglutinin is a sialic acid receptor-binding molecule and mediates entry of the virus into the target cell. The neuraminidase inhibitor (and oseltamivir and zanamivir) block viruses release via cleaving the cellular-receptor sialic acid residues to which the newly formed particles are attached (Moscona 2005). If the infection is limited to one round of replication there are not enough virus particles to cause disease. However, dapivirine, an FDA-approved HIV non-nucleoside reverse transcriptase inhibitor, was found to have broad-spectrum antiviral activity against multiple strains of influenza A and B viruses (Hu et al., 2017).

### Coronavirus

SARS-CoV and Middle East Respiratory Syndrome (MERS)-CoV are known as causative agents associated with high case fatality rate, whereas the other four human coronaviruses (HCoV-NL63, HCoV-229E, HCoV-OC43, and CoV-HKU1) are mainly associated with mild, self-limiting respiratory illnesses in immunocompetent hosts. Recently, a highly contagious agent that has emerged in China, SARS-CoV-2, was incidentally discovered in the case of cluster persons with acute respiratory infection identified whose clinical features resembled those of a viral pneumonia. Together with the above coronaviruses, SARS-CoV-2 account for a global threat to public health. Coronavirus belongs to the Coronaviridae family within the order of *Nidovirale*, contains a nonsegmented, positive-sense RNA genome of approximately 30 kilobase (kb). Coronavirus has currently been subdivided into four groups—the alpha-, beta-, gamma-, delta—on the basis of phylogenetic clustering (Fehr and Perlman, 2015; Kang et al., 2020). A canonical set of four main proteins of coronavirus virions are the spike (S), membrane (M), envelope (E), and nucleocapsid (N) protein, of which the first three are located in membrane envelope and the last one found in the ribonucleoprotein. Several crystal structures have been determined for coronaviruses, and these provide attractive targets for antiviral drug design. Here, we will focus on coronaviruses infection to human host and three coronavirus SRAS, MERS, and COVID-19 are the keystone to expound.

#### Human Coronaviruses

Prior to 2013, HCoV strains were primarily considered possible etiological agents in CAP that replicate in the epithelial cells of the nasopharynx and induce human illnesses, not only in the common colds but also in pneumonia (Hendley et al., 1972; Kunkel and Herrler, 1993; Vabret et al., 2003). Two of human coronaviruses are classified as α-coronaviruses, HCoV-229E and HCoV-NL63, while the others are β-coronaviruses, HCoV-HKU1 and HCoV-OC43. HCoVs provide significant insights into the genetic variability and evolution among coronaviruses. HCoV-NL63 displays homology with HCoV-229E based on phylogenetic analyses (Pyrc et al., 2007). A study found that the significantly greater association of HCoVs coinfections shows a rate of severe lower respiratory tract infections greater than 60% in patients with coinfections compared to less than 10% in patients with a single infection, especially in neonates and young children, although whether the coinfection by HCoV was a factor increasing the severity of the associated viral infection remains hypothetical (Gerna et al., 2006). Most HCoV infections are not diagnosed because they cause mild, self-limited upper respiratory disease, and no specific therapy is available.

#### Highly Pathogenic Disease: SARS, MERS, and COVID-19

Modern society did not draw high attention to coronavirus until the SARS-CoVs outbreak. During the 2002–2003 SARS pandemic, there were 8,422 cases of SARS-CoV in 32 countries, with 916 deaths and a fatality rate of 10–15% (WHO 2003). A novel human CoV, named MERS-CoV, emerged in the Middle East in 2012, and by October 16, 2018, 2,260 confirmed cases of infection with MERS-CoV had been documented in 27 countries by the WHO and were associated with 803 deaths (WHO 2020a; Kim et al., 2021).

SARS-CoV and MERS-CoV originated from bats that infected other intermediary reservoir in closer proximity to humans. It is widely accepted that SARS-CoV stems from a number of cave-dwelling species of Chinese horseshoe bats (genus *Rhinolophus*) (Li et al., 2005a; Lau et al., 2005). Both of them are β-coronavirus that mainly invade type II pneumocytes and bronchial epithelial cells, resulting in pneumonia, but the exact mechanism of lung injury is controversial. The highly glycosylated spike protein (S) host–receptor interaction plays a major determinant of initiating virus entry into the host cells. Human angiotensin-converting enzyme 2 (ACE2) binding is a critical determinant for the host range of SARS-CoV, whereas MERS-CoV utilizes dipeptidyl peptidase 4 (DPP4) as a cellular receptor, also known as CD26 (Bang et al., 2016). This carbohydrate shield may act as a target for compounds specifically binding to sugar moieties (e.g., lectins), accordingly coating the protein and blocking the interaction with the receptor (Mbae et al., 2018).

Up to now, neither approved specific drugs nor monoclonal antibody therapies to treat these two kinds of coronavirus infections. It is well known that three cysteine proteases, papain-like protease (PL^pro^) and 3C-like protease (3CL^pro^), as well as RNA-dependent RNA polymerase (RdRp), are validated antiviral drug targets because they are the components of the coronavirus lifecycle that mediate the replicase polyproteins pp1a and pp1b (Kim et al., 2014). Although the primary functions of PL^pro^ and 3CL^pro^ are to process the viral polyprotein in a coordinated manner, PL^pro^ has the additional function of stripping ubiquitin and ISG15 from host-cell proteins to aid coronaviruses in their evasion of the host innate immune response. Redemsivir (GS-5734) is a promising nucleotide analogue antiviral drug developed by Gilead science (Gordon et al., 2020). The HIV protease inhibitors approved by FDA, lopinavir and ritonavir (LPV/r) compound, were thought to markedly decrease the mortality of MERS-CoV or SARS (Chan et al., 2003; De Wilde et al., 2014; Sheahan et al., 2020). HR2P peptides may target the early stage of virus entry, namely the fusion between the envelope and cell membranes, which is supported by the evidence that HR2P is a highly efficient depressor for MERS-CoV S protein-mediated cell–cell fusion and syncytium formation (Lu et al., 2014).

The virus mutation is more likely to develop severe complications from coronavirus pneumonia that requires strengthened clinical vigilance. Currently, COVID-19 has been a challenge to global public health. A novel coronavirus, named SARS-CoV-2, was discovered by deep sequencing analysis from lower respiratory tract samples. It shows that SARS-CoV-2 can bind to ACE2 receptor in humans through structural analysis (Lu et al., 2020). The future evolution, adaptation, and spread of this virus warrant urgent investigation. LPV/r is also the first anti-HIV-1 drug reported to be tried for clinical treatment of SARS-CoV-2 infection. At this time, preventive therapies for these types of novel coronaviruses are still in preclinical stages.

## Natural Products With Reported Activities Against Viral Pneumonia: Focus on Medicinal Plants

**FIGURE 1:**
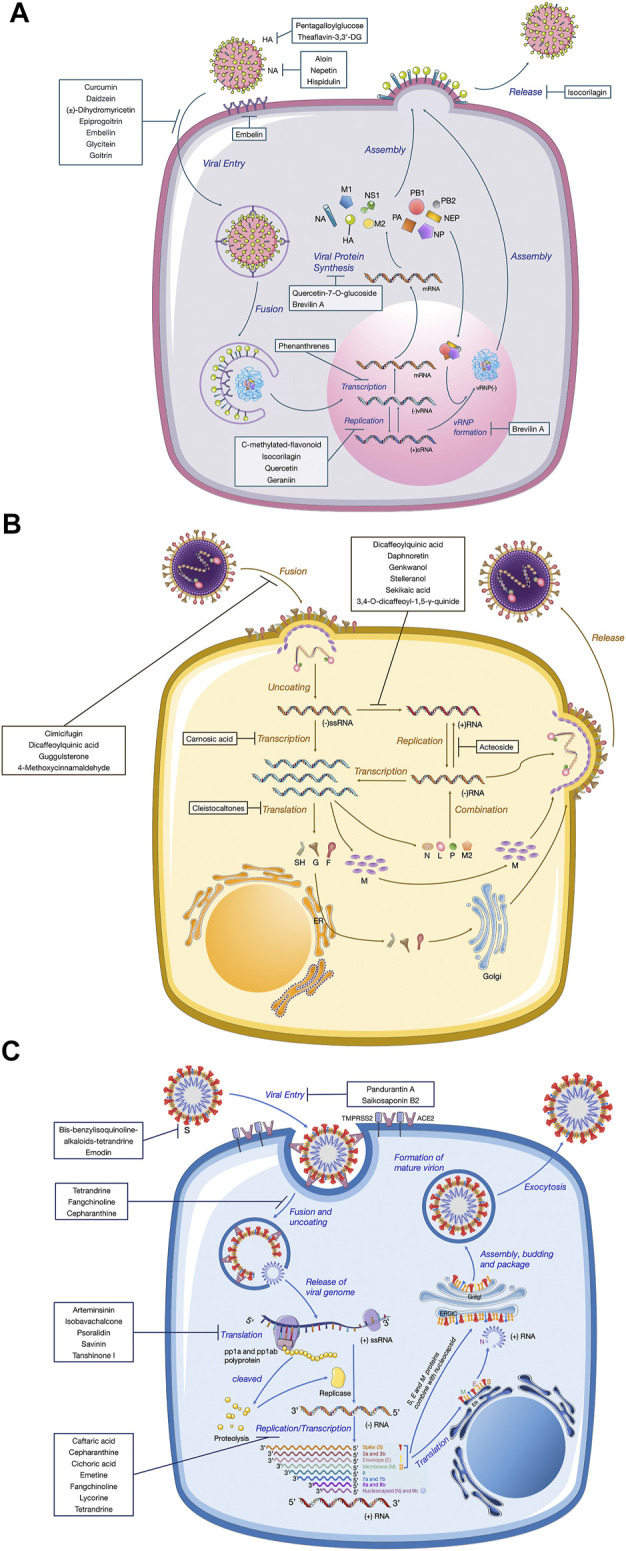
Replication mechanisms of three different kinds of virions. A diagram of the viral lifecycle of **(A)** influenza virus, **(B)** respiratory syncytial virus, and **(C)** coronavirus, indicating where each therapeutic exerts antiviral activity. The therapeutic targets of natural products derived from medicinal plants are shown in bold.

The recent emergence of the deadly human coronavirus that causes COVID-19 is a sobering reminder that new and deadly coronaviruses can emerge at any time and subsequently develop to become pandemics. Therefore, the continued development of therapeutic and prophylactic countermeasures to potentially deadly coronaviruses is warranted. At present, numerous bioactive constitutes targeted IFV, RSV and coronavirus have been screened and identified in the amelioration or prevention investigations of viral pneumonia. The screening procedure involves testing dilutions of the compounds against a range of viruses growing in cell cultures. Assays that a compound interferes with the proliferation of a virus might include inhibition of cytopathic effect (CPE) or of plaque formation. Selectivity and mechanism are crucial for the clinical use of antiviral drugs. Biologically, the underlying value of a compound can be assessed by the selectivity index (SI), which depends on its cytotoxic effect and antiviral activity. A compound with a low IC_50_ and a high SI is most likely to have a value as an anti-viral drug. Some cases of IC_50_ and SI values are given in Tables 1–4. Furthermore, the infection mechanism of these three viruses and modes of action of bioactive phytochemicals on them were shown in Figure 1.

**TABLE 1:** Medicinal plants extracts against influenza virus.

Plant species	Compound name	Extract	Strain, Subtype	Bioactivity	Assay	IC50/ EC50	SI	Positive control	References
*Aloe* *vera*(L.) Burm.f.	Aloin	Purchased from SA	A/PR/8/34 (H1N1)	Inhibited viral neuraminidase activity	Plaque	IC50: 91.83 ±18.97 μM (average value of all the tested strains)	>5.44	OTC: 25 μM	Huang et al. (2019)
			A/WSN/33 (H1N1)						
			A/TW/3446/02 (H3N2)						
			B/TW/70,555/5 (influenza B)						
			A/TW/126/09 (H1N1pdm09)						
			A/TW/066/09 (H1N1pdm09)						
*Burkea africana* Hook.	Oleanane-type triterpene saponins 7	Ethanol	A/Jena/8178/09 (H1N1pdm09)	Inhibited the HA (a hypothesis without verification)	CPE	IC50: 0.27 ± 0.13 μM	6	OTC: 0.064 ± 0.013 μM	Mair et al. (2018)
			A/Hong Kong/68 (H3N2)			IC50: 0.05 ± 0.02 μM	31	OTC: 0.003 ± 0.001 μM	
*Bletilla* *striata*(Thunb.) Rchb.f.	Phenanthrenes (analogs 4)	95% Ethanol	A/Sydney/5/97 (H3N2)	Inhibited matrix protein and reduced mRNA transcription; inhibited the NA	MTS	IC50: 14.6 ± 2.4 μM	5.5	OTC: 4.9 ± 0.9 μM	Shi et al. (2017a)
*Canarium album* (Lour.) DC.	Isocorilagin	75% Ethanol	A/Puerto Rico/8/34 (H1N1)	Interfered with replication; inhibited NA; influenced the virus release	MTT	IC50: 9.19 ± 1.99 μM	28.65	Peramivir: 6.48 μM	Chen et al. (2020)
			NA-H274Y (H1N1)			IC50: 4.64 ± 3.01 μM	56.75		
			A/Aichi/2/68 (H3N2)			IC50: 23.72 ± 2.51 μM	11.10		
*Centipeda* *minima*(L.) A. Braun and Asch.	Brevilin A	Supercritical fluid	A/PR/8/34 (H1N1)	Inhibited vRNA synthesis; decreased the M and NS protein expression	Plaque	EC50: 2.96 ± 1.10 μM	8	Ribavirin: 7.05 ± 1.10 μM	Zhang et al. (2019)
			A/FM/1/47 (H1N1)			EC50: 1.60 ± 1.14 μM	14	Ribavirin: 9.19 ± 1.02 μM	
			A/HongKong/498/97 (H3N2)			EC50: 3.28 ± 1.09 μM	7	Ribavirin: 10.76 ± 1.07 μM	
			A/chicken/Guangdong/1996 (H9N2)			EC50: 2.07 ± 1.12 μM	11	Ribavirin: 10.35 ± 1.04 μM	
*Camellia sinensis* (L.) Kuntze.	Theaflavin-3,3'-DG	Polyphenolic	A/PR/8/34 (H1N1)	Inhibited the NA and HA; decreased IL-6 expression	MTS	IC50:26.25 ± 6.20 μM	5.46	OTC:15.57 ± 1.73 nM	Zu et al. (2012)
			A/Sydney/5/97 (H3N2)			IC50: 10.67 ± 0.31 μM	13.43	OTC :8.88 ± 2.17 nM	
			B/Jiangsu/10/2003			IC50:42.07 ± 2.16 μM	3.41	OTC :31.60 ± 2.88 nM	
*Cleistocalyx operculatus* (Roxb.) Merr. and L. M. Perry	C-methylated- flavonoid	Methanol	A/PR/8/34 (H1N1)	Inhibited viral replication; affected an early stage of virus infection	Ez-Cytox	EC50: 4.9 ±0.35 μM	>24.49	Tamiflu: 2.24 ±0.15 μM	Dao et al. (2010)
*Curcuma longa* L.	Curcumin	Purchased from SA	A/PR/8/34 (H1N1)	Interrupted virus-cell attachment	Plaque	EC50: 0.47 ± 0.05 μM	92.5	-	Chen et al. (2020)
*Dianthus superbus* L.	Crude extract (quercetin and isorhamnetin were main compounds)	Butanol	A/PR/34/8 (H1N1)	Blocked viral replication	SRB	IC50: 4.97 ± 0.6 μg/ml	20.1	-	Kim et al. (2019b)
			B/LEE/40 (influenza B)			IC50:3.9 ± 0.5 μg/ml	25.4		
*Dianthus* *superbus* var.*longicalycinus*(Maxim.) F.N. Williams	Quercetin-7-O-glucoside	Methanol	A/PR/8/34 (H1N1)	Reduced virus-induced symptoms; blocked viral RNA polymerase PB2	SRB	IC50: 3.1 ± 0.43 μg/ml	32.35	OTC: 25.4 μg/ml	Gansukh et al. (2016)
			A/Vic/3/75 (H3N2)			IC50: 6.61 ± 0.08 μg/ml	15.19	OTC: 22.3 μg/ml	
			B/Lee/40			IC50: 8.19 ± 1.14 μg/ml	12.21	OTC: 42.2 μg/ml	
			B/Maryland/1/59			IC50: 5.17 ± 0.10 μg/ml	19.34	OTC: 35.2 μg/ml	
Embelia ribes Burm. f.	Embelin	Ethyl acetate	A/Puerto Rico/8/34 (H1N1)	Prevented absorption; blocked the cell receptors	MTT	IC50: 0.3±0.1 μM	10	OTC: 0.16±0.01 μM	Hossan et al. (2018)
			B/Malaysia/2506/04 (Victoria-like)			IC50: 0.2±0.1 μM	15	OTC: 0.31±0.04 μM	
			A/mallard/Pennsylvania/10218/84 (H5N2)			IC50: 0.1±0.0 μM	31	OTC: 0.1±0.02 μM	
*Glycine*max(L.) Merr.	Daidzein Glycitein	Water	A/PR/8/34 (H1N1)	Inhibited viral adsorption and replication	MTT	IC50: 143.6 ± 78.9 μM	>27	OTC: 0.628 nM	Nagai et al. (2019)
						IC50: 204.7 ± 21.0 μM	>17,182		
*Geranium* *thunbergii*Siebold ex Lindl. and Paxton.	Geraniin	Ethanol	A/PR/8/34 (H1N1)	Restricted viral replication	MTS	IC50: 27.6 μM	-	OTC: 0.628 nM	Choi et al. (2019)
			A/Korea/33/2005 (H1N1)			IC50: 11.1 μM		OTC: 0.338 nM	
			A/Korea/32/2005 (H3N2)			IC50: 25.8 μM		OTC: 0.855 nM	
			B/Korea/72/2006 (influenza B)			IC50: 8.72 μM		OTC: 10.8 nM	
*Isatis indigotica* Fortune ex Lindl.	Epiprogoitrin Progoitrin Epigoitrin Goitrin	Methanol	A/California/7/2009 (H1N1)	Disturbed viral adsorption	CCK8	IC50: 0.44 ± 0.03 μM	-	-	Nie et al. (2020)
						IC50: 0.19± 0.01 μM			
						IC50: 0.36 ± 0.02 μM			
						IC50: 0.19± 0.02 μM			
Not mentioned	Berberine-piperazine derivatives (analogs BPD-13)	Sythesis	A/PR/8/34 (H1N1) A/Vic/3/75 (H3N2) B/Lee/40 B/Maryland/1/5	Inhibited the NA	SRB	IC50: 35.16 ± 0.002 μg/ml	110.65 117.29 123.98 133.01	OTC: 21.12 ± 0.12 μg/ml	Enkhtaivan et al. (2018)
						IC50: 33.15 ± 0.021 μg/ml		OTC: 31.75 ± 0.55 μg/ml	
						IC50: 31.35 ± 0.031 μg/ml		OTC: 72.32 ± 0.066 μg/ml	
						IC50: 29.17 ± 0.081 μg/ml		OTC: 65.18 ± 0.037 μg/ml	
*Paeonia* *albiflora*Pall.	Pentagalloylglucose	Ethanol	A/PR/8/34 (H1N1)	Reduced the activity of virus NA and HA	MTT	IC50: 30.6 μM	27.4	OTC:100 μM	Zhang et al. (2019)
			A/WSN/33 (H1N1)			IC50: 20 μM	42 24		
			A/Hong Kong/1/68 (H3N2)			IC50: 34.8 μM	1		
*Portulaca oleracea* L.	Crude extract	Water	A/WSN/1933 (H1N1)	Inhibited viral attachment	MTS	EC50: 220.1μg/mL	36.65	-	Li et al. (2019)
			A/California/07/2009 (H1N1)			EC50: 121.6μg/mL	66.34		
			A/Perth/16/2009 (H3N2)			EC50: 112.4μg/mL	71.77		
			A/Brisbane/10/2007 (H3N2)			EC50: 191.2μg/mL	42.19		
*Rhodiola rosea* L.	Kaempferol	95% Methanol	A/PR/8/34 (H1N1)	Inhibited the NA	MTT	EC50: 30.2 μM	>9.93	Tamiflu: 8.3 μM	Jeong et al. (2009)
			A/Chicken/Korea/MS96/96 (H9N2)			EC50: 18.5 μM	>16.22	Tamiflu: 6.25 μM	
*Salvia* *plebeia*R.Br.	Nepetin Hispidulin Rosmarinic acid methyl ester	Methanol	A/PR/8/34 (H1N1)	Inhibited the NA	MTT	EC50: 17.45 ± 0.54 μM EC50: 22.62 ± 1.79 μM EC50: 22.60 ± 2.76 μM	11.47 ± 0.37 > 8.90 ± 0.76 8.98 ± 1.23	OTC: 0.10 ± 0.02 μM	Bang et al. (2016)
*Sambucus nigra* L.	5,7,3’,4’-Tetra-O-methyl quercetin (±)-Dihydromyricetin	Supercritical CO2; 80% Ethanol	A/PR/8/34 (H1N1)	Bound to the viral envelope; inhibited attachment	MTT	IC50: 0.36 μM	-	OTC: 0.32 μM	Roschek et al. (2009)
						IC50: 8.7 μM		Amantadine: 27 μM	
*Vitis amurensis* Rupr.	Amurensin K (+)-viniferol C Trans-vitisin B	Methanol	A/California/08/2009 (H1N1) A/PR/8/34 (H1N1) H274Y mutant (Oseltamivir-resistant novel H1N1)	Suppressed the activity of influenza NA	4-MU-NANA	IC50: 14.43±1.67 μM	-	OTC: 70.88±2.90 nM	Nguyen et al. (2011)
						IC50: 8.94 ± 1.06 μM		OTC: 3.89±0.75 nM	
						IC50: 23.89 ± 2.76 μM		OTC: 12.50 ± 0.56 μM	

**TABLE 2:** Medicinal plants extracts against respiratory syncytial virus.

Plant species	Compound name	Extract	Strain, Subtype	Bioactivity	Assay	IC50/ EC50	SI	Positive control	References
*Agastache* *rugosa*(Fisch. and C.A. Mey.) Kuntze	4-Methoxycinnamaldehyde	Purchased from WAKO	Long strain	Inhibited viral attachment and internalization; increased IFN production	XTT	IC50: 0.055 μg/ml	898.2	Ribavirin : 0.3-30 μg/ ml	Wang et al. (2009)
*Celastrus hindsii* Benth.	2α-hydro xyabietatriene	95% Ethanol	A2 strain	Not investigated	CPE	IC50: 3.13 ± 0.90 μM	-	Ribavirin: 4.1 ± 0.66 μM	Luo et al. (2018)
	Celahin D					IC50: 1.55 ± 0.34 μM			
	Vitamin E quinone					IC50: 3.13 ± 0.43 μM			
*Cimicifuga foetida* L.	Cimicifugin	Purchased from Sigma-Aldrich	Long strain (in A549 cells)	Inhibited viral attachment; stimulated epithelial cells to secrete IFN-β to counteract viral infection	XTT	IC50: 5.4 μg/ ml	45.57	Ribavirin: 29.8 μg/ ml	Wang et al. (2012)
			Long strain (in HEp-2 cells)			IC50: 38.6 μg/ml	6.48	Ribavirin: 31.8 μg/ ml	
*Cleistocalyx operculatus* (Roxb.) Merr&L.M. Perr*y*.	Cleistocaltones A	95% Ethanol	A2 strain	Reduced F proteins	MTT	IC50: 6.75 ± 0.75 μM	>14.81	Ribavirin: 15.00 ± 1.00 μM	Song et al. (2019)
	Cleistocaltones B					IC50: 2.81 ± 0.31 μM	9.02		
*Clerodendrum trichotomum* Thunb.	Acteoside	Water	rgRSV strain	Reduced replication; blocked syncytial formation	CCK8	EC50: 15.64 ± 1.07 ng/ ml	47.33	-	Chathuranga et al. (2019)
*Coffea arabica* L. (the source of synthetic materials)	3,4-O-dicaffeoyl-1,5-γ-quinide	Synthesis	Long strain 18537	Inhibited intracellular post-entry replication step	CPE	EC50: 0.240 μM	>416	Ribavirin: 5.05 μM	Sinisi et al. (2017)
			strain			EC50: 0.236 μM	>423		
			rgRSV strain			EC50: 0.170 μM	>588		
*Commiphora gileadensis* (L.) C. Chr.	Guggulsterone	Methanol	RSV B	Inhibited viral absorption	MTT	IC50: 23.31 μg/ml	10.25	-	Bouslama et al. (2019)
*Delphinium ajacis* L.	Ajacisine E	95% Ethanol	A2 strain	Not investigated	MTT	IC50: 10.1 ± 0.3μM	>9.9	Ribavirin:3.1 ± 0.8 µM	Yang et al. (2017)
*Euphorbia jolkinii* Boiss.	Jolkinol A	Methanol	Long strain	Not investigated	CPE	IC50: 10 μM	8	Ribavirin: 6.97 μM	Huang et al. (2014)
*Ficus religiosa* L.	Bark crude extract	Water	A2 strain	Inhibited viral attachment	MTS	EC50: 2.23 μg/ml	84.5	Ribavirin: 6.67 μg/ ml	Cagno et al. (2015)
*Forsythia* *suspensa* (Thunb.) Vahl.	Calceolarioside B	Ethanol	Not described	Not investigated	CPE	EC50: 3.43 μM	56.33	-	Li et al. (2014)
	Forsythoside A					EC50: 6.72 μM	34.23		
*Lonicera* *japonica* Thunb.	Dicaffeoylquinic acid	Ethanol	Long strain	Reduced virus replication and fusion	MTS	EC50:0.068 ± 0.002 μM	>5800	Ribavirin: 3.2 μM	Ojwang et al. (2005)
*Lophatherum gracile* Brongn.	Isoorientin	95% ethanol	Long strain A2 strain	Triggered inflammatory reactions; inhibited replication	MTT	IC50: 3.1 ± 0.2 μg/ ml	138.7	Ribavirin: 1.6 ± 1.0 μg/ ml	Chen et al. (2019)
*Narcissus* *tazetta* var.*algirus* (Pomel) Batt.	Narcissus tazetta lectin	-	Long strain	Bound to viral glycoproteins; affected the later infection phase	MTT	IC50: 2.3 μg/ ml	141.36	-	Ooi et al. (2006)
*Rosmarinus officinalis* L.	Carnosic acid	70% Ethanol	A2 strain (in A549 cells)	Inhibited NS2 and G protein RNA synthesis; affected viral factors	MTT	IC50: 6.51 μg/ ml	20.09	-	Shin et al. (2013)
			A2 strain (in HEp-2 cells)			IC50: 6.71 μg/ ml	-		
			B/KR strain			No numerical value	-		
*Schefflera* *heptaphylla* (L.) Frodin.	3,5-Di-O-caffeoylquinic acid 3,4-Di-O-caffeoylquinic acid	60% Ethanol	Long strain	Blocked virus-cell fusion; inhibited replication cycle at the late phase	MTT	IC50: 1.16 μM	1116	Ribavirin: 36.7	Li et al. (2005b)
						IC50: 2.23 μM	500		
*Smilax glabra* Roxb.	Mannose-binding lectin	Saline	Not described	Not investigated	CPE	EC50: 8.1 μM	-	Ribavirin:12.5 μM	Ooi et al. (2006)
*Wikstroemia indica* (L.) C. A. Mey.	Daphnoretin	Ethanol	Long strain	Reduced the PKC pool to affect fusion; inhibited viral replication at the later infection phase	MTT	IC50: 5.87 μg/ ml	28.17	Ribavirin: 3.05 μg/ ml	Ho et al. (2010)
*Wikstroemia indica* (L.) C. A. Mey.	Genkwanol B Genkwanol C Stelleranol	Ethanol	Long strain	Inhibited viral replication cycle	MTT	IC50: 9.6 μM	11.0	Ribavirin: 21.6 μM	Huang et al. (2010)
						IC50: 6.6 μM	21.9		
						IC50: 10.2 μM	15.8		
*Wikstroemia indica* (L.) C.A.Mev	Sekikaic acid	Ethyl acetate	rgRSV strain A2 strain	Inhibited viral replication at a post-entry step	MTT	IC50: 5.69 μg/ ml	5.46	Ribavirin: 1.20 ± 0.45 μg/ ml -	Lai et al., (2013)
						IC50: 7.73 μg/ ml	4.02		
*Youngia japonica* (L.) DC.	Dicaffeoylquinic acid	95% Ethanol	Long strain	Affected the early stage of viral replication	CPE	IC50: 0.5 μg/ ml	>200	Ribavirin: 2.5 μg/ ml	Ooi et al. (2006)

**TABLE 3:** Medicinal plants extracts against coronavirus.

Plant species	Compound name	Extract	Cell lines	Strain, Subtype	Bioactivity	Assay	IC50/ EC50	SI	Positive control	References
*Alnus japonica* (Thunb.) Steud.	Hirsutenone	Ethanol	-	SARS-CoV	Inhibit PL^pro^	RLRGG-AMC	IC50: 4.1±0.3 μM	-	-	Park et al. (2012a)
*Artemisia* *carvifolia* Buch. -Ham. ex Roxb.	Arteether	Purchased from market	Vero E6	SARS-CoV-2	Reduced viral NP protein; blocked viral infection at the post-entry level; inhibited viral RNA and protein	CCK8	EC50: 31.86 ± 4.72 μM	>6.42 ± 0.95 >3.13 ± 1.14 >3.11 ± 0.12 >4.03 ± 0.15 =5.10 ± 2.08 =7.00 ± 0.76 >4.40 ±0.61	-	Cao et al. (2020)
	Artemether						EC50: 73.80 ± 26.91 μM			
	Arteminsinin						EC50: 64.45 ± 2.58 μM			
	Artmisone						EC50: 49.64 ± 1.85 μM			
	Artesunate						EC50: 12.98 ± 5.3 μM			
	Lumefantrine						EC50: 10.28 ± 1.12 μM			
	Arteannuin B						EC50: 23.17 ± 3.22 μM			
*Boesenbergia* *rotunda* (L.) Mansf.	Pandurantin A	95% EtOH	Vero E6	SARS-CoV-2	Inhibited at both pre‐entry and postinfection phase	MTT	IC50: 0.81 μM	18.16	-	Kanjanasirirat et al. 2020,
	Hydroxychloroquin						IC50: 5.08 μM	>19.68		
*Bupleurum chinense* DC.	Saikosaponin A	Purchased from Sigma Chemical	MRC-5	HCoV-229E	Inhibited viral attachment	XTT	EC50: 8.6 ± 0.3 μM	26.6	Actinomycin D: 0.02 μM	Cheng et al. (2006)
	Saikosaponin B_2_						EC50: 1.7 ± 0.1 μM	221.9		
	Saikosaponin C						EC50: 19.9 ± 0.1 μM	19.2		
	Saikosaponin D						EC50: 13.2 ± 0.3 μM	13.3		
*Cornus officinalis* Siebold and Zucc.	Savinin Betulonic acid	Ethyl acetate	Vero E6	SARS-CoV	Inhibited PL^pro^ and 3CL^pro^	MTT	EC50: 1.13 μM	>667	Niclosamide: <0.1 μM *Valinomycin: 1.63* *μM*	Wen et al. (2007)
							EC50: 0.63 μM	180		
*Echinacea* *purpurea* (L.) Moench.	Standardized preparation (caftaric acid and cichoric acid were main compounds)	65% Alcoholic	Vero E6	HCoV-299E	Interacted with virions; inhibited replication	MTT	IC50: 3.2 μg/ml	-	-	Signer et al. (2020)
*Euphorbia neriifolia* L.	3β-friedelanol	Ethanol	MRC-5	HcoV-229E	Induced cell death	XTT	5 μg/ml	-	0.02 μg/ml	Chang et al. (2012)
*Forsythia suspensa* (Thunb.) Vahl	Phillyrin	Purchased from market	Vero E6 Huh-7	SARS-CoV-2 HcoV-229E	Regulated host immune response	MTT	IC50: 63.90 μg/ml	30.66	-	Ma et al. (2020)
							IC50: 64.53 μg/ml	16.02		
*Glycyrrhiza* *uralensis* Fisch.	Glycyrrhizic acid	Purchased from commercial suppliers	MASMCS 16HBE	SARS-CoV-2 S protein	Blocked the binding between the RBS and ACE2	MTT SPR	IC50: 22 μM	>4.55	-	Yu et al. (2020)
*Glycyrrhiza uralensis* Fisch.	Glycyrrhizin	Purchased from market	Vero E6	SARS-CoV	Induced nitrous oxide synthase and viral replication	MTT	EC50: 300±51 mg/L	>67	Pyrazofurin: 4.2 ± 0.57 mg/L 6-azauridine: 16.8 ± 2.9 mg/L	Cinatl et al. (2003)
*Lithospermum* *erythrorhizon* Siebold and Zucc.	Shikonin	Not described	Vero E6	SARS-CoV-2	Inhibited 3CL^pro^	CCK8	IC50:15.75 ± 8.22 μM	-	Ebselen: 0.67 ± 0.09 μM	Jin et al. (2020)
*Lycoris* *radiata* (L’Hér.) Herb.	Lycorine	Purchased from MicroSource Discovery Systems	LLC-MK2	HCoV-OC43	Suppressed viral replication	MTT	EC50: 0.15 μM	29.13	-	Shen et al. (2019)
Not mentioned	Resveratrol	Purchased from Sigma-Aldrich	Vero E6	HcoV-229E SARS-CoV-2	Not investigated	MTT	EC50: 4.6 μM	45.65	Lopinavir/ritonavir: 8.8 μM Chloroquine: 5.0 μM	Pasquereau et al. (2021)
							EC50: 10.66 μM	4.52		
*Polygonum cuspidatum* Siebold and Zucc.	Resveratrol	Not described	Vero E6	MERS-CoV	Prolonged cellular survival; inhibited replication targeted N; blocked NF-κB pathway	MTT	125-250 μM	-	-	Lin et al. (2017)
*Psychotria* *ipecacuanha* (Brot.) Standl.	Emetine	Purchased from MicroSource Discovery Systems	Vero E6	MERS-CoV	Inhibited viral entry and replication	MTT	EC50: 0.34 μM	9.06	-	Shen et al. (2019)
*Psoralea corylifolia* L.	Isobavachalcone	Ethanol	-	SARS-CoV	Inhibited PL^pro^	Z-RLRGG-AMC	IC50: 7.3 ± 0.8 μM	-	-	Kim et al. (2014)
	Psoralidin						IC50: 4.2 ± 1.0 μM	-		
*Rheum* *palmatum* L.	Emodin	Water	Vero E6	SARS-CoV	Inhibited the interaction of viral S protein and cell receptor ACE2	MTT	IC50: 200 μM	-	Promazine	Ho et al. (2007)
*Sambucus* *formosana* Nakai.	Caffeic acid	Purchased from Sigma-Aldrich	LLC-MK2	HcoV-NL63	Blocked viral attachment; Inhibited viral replication	MTT	IC50: 3.54 μM	>141	-	Weng et al. (2019)
*Salvia miltiorrhiza* Bunge.	Tanshinone I	Ethanol	-	SARS-CoV	Inhibited PL^pro^	LXGG-AMC	IC50: 0.7 μM	-	-	Park et al. (2012b)
*Stephania tetrandra* S. Moore	Bis-benzylisoquinoline alkaloids-tetrandrine	Purchased from Wuhan ChemFaces Biochemical	MRC-5	HcoV-OC43	Suppressed viral replication; inhibited S and N protein	MTS	IC50: 0.33 ± 0.03 μM	40.19	-	Kim et al. (2019a)
	Fangchinoline						IC50: 1.01 ± 0.07 μM	11.46		
	Cepharanthine						IC50: 0.83 ± 0.07 μM	13.63		

**TABLE 4:** Molecular docking results of influenza virus, respiratory syncytial virus, and coronavirus.

Plant species	Compound name	Virus Type	Binding Subunit	PDB Code	Affinity (kcal/mol)	Residues	Positive(kcal/mol)	Software	References
*Anethum graveolens* L.	Quercetin	SARS-CoV-2	3CLpro	6LU7	−8.17−8.47	His164, Glu166, Asp187, Gln192, Thr190	Nelfinavir : −10.72 Lopinavir : −9.41	Autodock 4.2	Khaerunnisa et al. (2020)
*Allium cepa* L. *Cocos nucifera* L.	Oleanolic acid Progesterone Stigmasterol Fucosterol	SARS-CoV-2	3CLpro	6W63	−9.2−8.4−9.4−9.1	Cys145, Met49, Met165, Leu167, Pro168 Gly143, Gln192, Thr190, Met165 Met165, Met49, Cys44, Cys145, His41 Met49, Met165, Leu167, Pro168, His41, Cys145	Remdesivir: -7.6	AutoDock 4.2	Fitriani et al. (2020)
*Camellia* *sinensis* (L.) Kuntze.	Thearbigin Quercetin-3-O-rutinoside	SARS-CoV-2	3CLpro	6LU7	−8.5−7.5	Glu166, Asn142, Met165, Cys145 Glu166, Leu141, Gly143, Asn142	-	ParDock	Upadhyay et al. (2020)
*Chrysanthemum cinerariifolium* (Trevir.) Sch.Bip.	Rutin Schaftoside Apigenin‐6,8‐di‐C‐β‐D‐galactoside	RSV	N	4UCC	−8.49−8.18 −7.29	Glu128, Glu112, Arg132, Asp152, Arg150 Glu144, Arg132, Glu128, Lys110, Asp152 Arg132, Glu112, Glu128, Lys110, Lys46	1-[(2,4-dichlorophenyl) methyl]	Maestro 9.3	Kant et al. (2018)
							pyrazole-3,5- dicarboxylic acid: -5.95		
*Coffea arabica* L. (the source of synthetic materials)	Berberine-piperazine derivatives	Influenza A Virus (H3N8)	NA	4WA4	−8.2	Ala432, Arg116, Arg150, Ser178, Ile221, Trp177	OTC: -6.1	AutoDock Vina	Enkhtaivan et al. (2018)
*Dianthus superbus* var. *monticola Makino*	Quercetin-7-O-glucoside	Influenza A Virus	PB2	4NCE	−9.1	Ser321, Ser324, Arg332, His342, Met431, Lys376, Glu361, His357, Phe323, Phe404	m^7^GTP: -7.5	AutoDock Vina	Gansukh et al. (2016)
*Dianthus superbus* L.	Quercetin 3-rutinoside Quercetin 3-rhamnoside 7-rhamnoside Kaempferol 3-glucoside-Glucoside 7-rhamnoside	Influenza A virus (pdmH1N109)	PA	4AWM	−9.8 −9.7 −8.9	Not described	-	AutoDock Vina	Kim et al. (2019b)
*Dianthus superbus* L.	Quercetin 3-glucoside	Influenza A virus (H3N2)	PB2	4NCE	−8.0	Arg355, Arg332, Lys376, Ile354, Met431, Phe323, Phe363, Phe404, Asn429	GTP: -7.0	AutoDock Vina	Nile et al. (2020)
*Embelia ribes* Burm.f.	Embelin	Influenza A Virus (H5N2)	HA	5E30	−5.2	Glu190, Arg193, Ser227, Gly228, Tyr98, Val135	α-2,6 linked terminal sialic acids (SAs): -6.2	AutoDock Vina	Hossan et al. (2018)
*Geranium thunbergia* Siebold ex Lindl. & Paxton	Geraniin	Influenza A Virus (H1N1pdm09)	NA	3TI6	−9.9	Arg225, Glu227	OTC: -6.6	AutoDock Vina	Choi et al. (2019)
*Glycyrrhiza uralensis* Fisch	Liquiritigenin	Influenza Virus	NA	4B7N	−7.05	Arg118, Ile149, Arg368, Ser400, Ile427, Pro431, Lys432	-	AutoDock	Sathya et al. (2019)
*Laminaria japonica* Aresch.	Dieckol	SARS-CoV	3CLpro	2ZU5	−11.51	Thr190, His63, Ser144, Cys145, His41	-	AutoDock 3.0.5	Park et al. (2013)
Not mentioned	Quercetin-3-β-galactoside	SARS-CoV	3CLpro	1UK4	−9.24	Leu141, Asn142, Met165, Glu166, Gln189	-	DOCK4.0	Cheng et al. (2006)
Not mentioned	Aloe-emodin + (-) Epicatechin Rhein Withanolide D Withanolide A	SARS-CoV-2	3CLpro	6LU7	−7.4 −7.6 −8.1 −7.8 −7.7	Not mentioned Not mentioned Ile106, Gln110, Thr29, Thr111, Phe294, Asp295 Lys102, Phe103, Val104, Arg105, Ile106 Phe294, Thr292, Asp295, Asp153, Ser158	Nelfinabir: -8.4	Swiss Dock	Chandel et al. (2020)
Not mentioned	Hypericin Cepharannthine	SARS-CoV	NSP12‐NSP8	6NUR	−8.3−7.9	-	Nilotinib: -8.4	Autodock Vina	Ruan et al. (2021)
							Tegobuvir: -8.4		
Not mentioned	Cepharanthine Hypericin Berberine	SARS-CoV-2	NSP12‐NSP8	7BW4	−8.6 −8.2 −7.8	Arg215, Leu155, Val225	-	AutoDock Vina	Ruan et al. (2021)
*Olea Europaea* L.	Luteolin-7-glucoside	SARS-CoV-2	3CLpro	6LU7	−8.17 −8.47	Phe140, Cys145, His163, His164, Thr190	Nelfinavir : -10.72	Autodock 4.2	Khaerunnisa et al. (2020)
							Lopinavir : -9.41		
*Radix Paeoniae* Alba.	Gallic acid	Influenza A virus (H1N1)	NA	3CKZ	−5.7	Arg152, Glu227	-	AutoDock Vina	Zhang et al., 2020
*Rapanea melanophloeos* (L.) Mez	Quercetin-3-O-α-L-rhamnopyranoside	Influenza Virus	M2 NA	2KQT 3TI6	−10.81 −10.47	Ala30, Ile33, Val27 Asp151, Asn347, Ile149	Rimantadine: -5.51	Glide	Mehrbod et al. (2019)
							OTC: -7.06		
*Spinacia oleracea* L.	Kaempferol	SARS-CoV-2	3CLpro	6LU7	−8.58	Tyr54, His164, Glu166, Apr187, Thr190	Nelfinavir : -10.72	Autodock 4.2	Khaerunnisa et al. (2020)
							Lopinavir : -9.41		
*Torreya nucifera* (L.) Siebold and Zucc.	Amentoflavone	SARS-CoV	3CLpro	2Z3E	−11.42	His163, Leu141, Gln189, Val186, Cys145, His41	Apigenin: -7.79	Autodock 3.0.5	Ryu et al., 2010b
*Tripterygium* *wilfordii*Hook. f.	Iguesterin	SARS-CoV	3CLpro	1UK4	−9.97	Cys44, Thr25	-	Autodock 3.0.5	Ryu et al. (2010a)
*Veratrum* *sabadilla* Retz.	Sabadinine	SARS-CoV	3CLpro	Not mentioned	−11.6	His44, Cys144	-	AutoDock Vina	Toney et al. (2004)

### Anti-Influenza Virus Agents

Recently, Nie et al. (2020) screened two pairs of enantiomers (glucosinolate isomers) isolated from *Isatis indigotica* Fortune ex Lindl. by chiral separation against influenza A virus (IAV), which among the antiviral potency of the components was in the order of progoitrin > goitrin > epigoitrin > epiprogoitrin. Elderberry (*Sambucus nigra* L.), a traditional European medicine rich in flavonoids, plays an essential role in anti-influenza and immune stimulation. Early stage of research established that two anti-influenza flavonoids isolated from elderberry fruit inhibit H1N1 infection by decreasing the ability of infecting host cells (Roschek et al., 2009). Nevertheless, quality assurance must be considered on account of unripe elderflower fruits contain a certain amount of sambunigrin, a latent toxic glycoside can release cyanide of which concentration decreases in the ripening process (Vlachojannis et al., 2010; Stuppner et al., 2020). Quercetin, a kind of natural compounds marketed as a dietary supplement, also exhibits a good performance of inhibiting virus, which frequently in daily doses of up to 1,000 mg d^−1^ exceeds usual dietary intake levels (Andres et al., 2018). An investigation carried out by Gansukh et al. elucidated that quercetin-7-O-glucoside might be useful in alleviating symptoms and pathogenesis in the host (Gansukh et al., 2016). In a later study the same team further confirmed the value to IFV of *Dianthus superbus* L. (Kim et al., 2019b). Quercetin 3-glucoside (Q3G), an analogous flavonoid obtained from the methanol extract of *D. superbus*, was the most active fraction in blocking IFV replication via a time-dependent assay (Nile et al., 2020). Specially, brevilin A and C-methylated flavonoid that were determined to be (E)-4,2′,4′-trihydroxy-6′-methoxy-3′,5′-dimethylchalcone have the similar bioactive mechanism as Q3G (Dao et al., 2010; Zhang et al., 2019).

Among plants-derived compounds, *Canarium album* (Lour.) DC. derived isocorilagin, a polyphenolic compound, showed an antiviral activity against diverse influenza A strains by targeting NA with low cytotoxic effects against host cells (Chen et al., 2020). Furthermore, *Canarium album* (Lour.) DC. is widely used as a medicinal and edible plant with characteristics of safe and economic. Taken together, isocorilagin promises to be a highly effective, reliable, and affordable neuraminidase inhibitor against a range of IAV strains. *Portulaca oleracea* L. water extract was able to alleviate the symptoms of pandemic IAV infection. Further mechanistic studies revealed that it clearly inhibited the virus-cells attachment and exerted good virucidal activity, significantly reducing the viral load within 10 min (Li et al., 2019). Geraniin displayed high antiviral activity against influenza A and B strains, by inhibiting NA activity following viral infection in MDCK cells (Choi et al., 2019). The OECD 423 acute oral toxicity test carried out by a team demonstrated that geraniin was safe for human consumption with the no-observed-adverse-effect level of geraniin being below 2000 mg kg^−1^, while that of geraniin-enriched extract was more than 2000 mg kg^−1^ (Moorthy et al., 2019). Embelin are well known for their antiviral properties exhibiting a strong inhibitory effect on influenza replication, in particular the strain B, with the lowest IC_50_ value of 0.2 ± 0.1 μM (Hossan et al., 2018).

A further example of a plant-derived natural product is nepetin, a methanolic extract originated from aerial parts of *Salvia plebeia* R.Br. provides noteworthy candidates for further investigation of novel NA inhibitors in the future (Bang et al., 2016). Huang et al. (2019) conducted a study to investigate the efficacy of aloin, which is contained in *Aloe vera* (L.) Burm.f., to reduce virus load in the lungs. Yet, it is worth noting that aloin was reported to be the most toxic in all the compounds of *A. vera*, and related institutions have introduced the safety usage guideline on recommending acceptable amounts of aloin in pharmaceuticals and food (Kaparakou et al., 2021). Since 2002, the FDA has suggested that it should not exceed 10 mg L^−1^ aloin in *A. vera* products when used as food or as a dietary supplement. As part of an ongoing anti-influenza screening project on natural products, eight oligostilbenes were isolated as active principles from the methanol extract of *Vitis amurensis* Rupr. among which (+)-viniferol C and amurensin K showed the excellent antiviral activity (Nguyen et al., 2011). Daidzein and glycitein, two active compounds isolated from Glycine max (L.) Merr., demonstrated excellent antiviral activity, among which the latter was firstly reported with anti-IFV effect (Nagai et al., 2019). Different compounds derived from *Burkea africana* Hook. and *Bletilla striata* (Thunb.) Rchb.f. have been identified for their role against H3N2 strains (Shi et al., 2017b; Mair et al., 2018). Moreover, *Rhodiola rosea* L. and black tea commonly used plants as ethnomedicine in China for infectious diseases, and exhibited IFV proliferation at low concentrations (Mukhtar et al., 2008; Jeong et al., 2009; Zu et al., 2012).

It shows a natural product derivative or conjugate against IFV in the overview. The anti-influenza activities of berberine-piperazine derivatives (BPD) were evaluated in the range from 35.16 μg/ml to 90.25 μg/ml of the IC_50_ along with cytotoxicity level which was observed in the range 44.8 μg/ml to 3,890.6 μg/ml of CC_50_ toward MDCK cells (Enkhtaivan et al., 2018).

### Anti-Respiratory Syncytial Virus Agents

Generally rational drug use for different CAPs is summarized in Table 2. Many medicinal plants have been reported to treat animals and people who suffer from RSV-related pneumonia. Caffeoylquinic acids (CQAs) are a broad class of secondary metabolites that have been found in esculent and medicinal plants from various families. Accumulated evidence demonstrated that CQAs have a wide range of biological activities including antiviral effects. One such example is dicaffeoylquinic acid, respectively isolated from *Lonicera japonica* Thunb. and *Youngia japonica* (L.) DC., which produced obvious anti-RSV activity that is better than that of ribavirin (Ojwang et al., 2005; Ooi et al., 2006). A caffeoylquinic acid derivative, namely 3,5-Di-Ocaffeoylquinic acid purified from Schefflera heptaphylla, could inhibit RSV with IC50 at 1.16 μM (Li et al., 2005b). Although CQAs have increased interest for using as antiviral therapeutics, the reports of their safety pharmacological effects are limited.

4-Methoxycinnamaldehyde, an active constituent of *Agastache rugosa* (Fisch. and C.A. Mey.) Kuntze, could suppress viral entrance by interfering viral attachment (IC_50_ of 0.06 mg/ml) and internalization (IC_50_ of 0.01 mg/ml). The compound significantly increased the basal production of IFN, but not the virus-induced IFN production (Wang et al., 2009). In a later study Wang’s team also found that a major compound of *Cimicifuga foetida* L., namely cimicifugin, possessed inhibitory activity against RSV through suppressing viral attachment and internalization (Wang et al., 2012). Yet for all that, the potential risk existing in administrating cimicifuga plants cannot be reckoned with, particularly the severe hepatotoxicity. It has been reported that in two cases patients developed fulminant hepatic failure due to the use of this herbal remedy (Levitsky et al., 2005; Chow et al., 2008). Therefore, safety consideration should be retained as a high priority for novel drugs cimicifuga therapeutics in the early stages of development and clinical trials (Guo et al., 2017). A compound 19 (jolkinii A) of *Euphorbia jolkinii* Boiss. displayed significant anti-RSV activity, with an IC_50_ value of 10 μM and an SI of 8.0 (Huang et al., 2014). In recent years, Chathuranga et al. (2019) also suggested that extracts of *E. jolkinii* could provide a potential source of antiviral candidate against RSV infection. In his investigation, oral inoculation with each herb extract obviously improved viral clearance in the lungs of BALB/c mice. Two novel phloroglucinol-terpenoid adducts (Cleistocaltones A and B) as the anti-RSV test compounds reduced the expression of RSV F proteins and showed IC_50_ values of 6.75 ± 0.75 μM and 2.81 ± 0.31 μM, respectively, and were isolated from the buds of *Cleistocalyx operculatus* (Roxb.) Merr. and Perry (Song et al., 2019).

A study of isoorientin provides a convincing and powerful support for the traditional use of *Lophatherum gracile* Brongn. in the RSV-related diseases treatment (Chen et al., 2019). Following the probit analysis of brine shrimp lethality assay, the LD_50_ values of isoorientin were calculated to be more than 1000 μg/ml compared to cytotoxic lignan podophyllotoxin with 2.79 μg/ml. It could be effective if isoorientin was to be treated as safe drugs, of which high LD_50_ values indicated very low general toxicity (Kumarasamy et al., 2004). Other glycosides, such as calceolarioside B, genkwanol C, were also discovered as anti-RSV agents (Huang et al., 2010; Li et al., 2014). Bouslama et al. (2019) have put forward a number of possible mechanisms whereby guggulsterone may exert their antiviral action. They suggested that the antiviral activity in guggulsterones probably derives from the steps involving recognition and binding to specific receptors. The antiviral composition of essential acid of *Rosmarinus officinalis* L. suppressed the replication of HRSV and viral gene expression without inducing type-I interferon production or affecting cell viability in viral suspension tests (Shin et al., 2013). Two lectins, mannose-binding lectin and narcissus tazetta lectin, were isolated from *Smilax glabra* Roxb. and *Narcissus tazetta* L., respectively. Both of them have the function of broad-spectrum antivirus, including RSV and IFV. The virus was also susceptible to a crude water extract (Saponins/Carbohydrate/Tannins) from *Ficus religiosa* L. bark (Cagno et al., 2015).

Three terpenoids 2α-hydroxyabietatriene, celahin D, and vitamin E quinone showed inhibitory activity on reverse transcriptase activity with an IC_50_ of approximately 3.13 μM. In the contrast, celahin D showed the lower one compared to three compounds (Luo et al., 2018). Further isolation of *Wikstroemia indica* (L.) C.A. Mey. fraction led to a purified compound, daphnoretin. It was found to have anti-RSV activity using CPE assay, with an IC_50_ value of 5.87 mg/ml and an SI value of 28.17 (Ho et al., 2010). *W. indica* is thought to be poisonous; there are adverse effects that can be caused, including dizziness, nausea, vomiting, and diarrhea. Therefore, too much inhalation and skin contact are not allowed when processing, grinding, and decocting (Li et al., 2009). Sheng-Ma-Ge-Gen-Tang (SMGGT) has been used to treat pediatric viral infection and one of the most effective medicine herbs is *Cimicifuga foetida* L., which could be useful for preventing and managing viral infection by stimulating IFN-β (Wang et al., 2012; Feng Yeh et al., 2013).

### Anti-Coronavirus Agents

There are several medicinal plants that treat for three kinds of HCoVs. Recently, the present results indicated that saikosaponin B_2_ and 3β-friedelanol both have potent natural drugs against HCoV-229E *in vitro* and that their modes of action possibly involve interference in the early stages of viral replication, such as absorption and penetration of the virus (Cheng et al., 2006; Chang et al., 2012). The bis-benzylisoquinoline alkaloids tetrandrine, fangchinoline, together with cepharanthine, which are particularly high in *Stephania tetrandra* S. Moore and other related species of Menispermaceae, dramatically suppressed the replication of HCoV-OC43 and inhibited expression of protein S and N (Kim et al., 2019a). One research showed that Sambucus javanica Blume. stem ethanol extract displayed potential anti-HCoV-NL63 activity; caffeic acid could be the vital component with anti-HCoV-NL63 activity via interfering the binding interaction of HCoV-NL63 with heparan sulfate proteoglycans (co-receptor) and ACE2 (receptor) on cell surface (Weng et al., 2019).

Renowned as polyphenolic phytoalexin with a wide range of biological properties, resveratrol (3,5,4’-trans-trihydroxystilbene) administration spans a large spectrum of areas, especially the prevention and treatment of viral diseases. It has been shown, in the present study against coronavirus, that resveratrol demonstrated the important impact on anti-HCoV-229E and anti-SARS-CoV-2 compared to LPV/r and chloroquine (Pasquereau et al., 2021). In addition, cohort clinical trials that document the efficacy, safety, and pharmacokinetics provided evidence that the side effects of resveratrol are mild and sporadic compared with its overwhelming health benefits (Sedlak et al., 2018; Galiniak et al., 2019; Singh et al., 2019). Therefore, resveratrol could be a promising candidate to further use in a clinical testing in fighting COVID-19.

As a pivotal enzyme of mediating replication and transcription in coronaviruses, 3CL^pro^ has become a magnet for new drugs target. Shikonin exhibited promising antiviral activity by targeting 3CL^pro^ (Jin et al., 2020). Nevertheless, it needs to be carefully used. In an acute toxicity study, the median lethal dose (LD_50_) was calculated to be 20 mg/kg in mice, while the median lethal concentration (LC_50_) was 16 mg/kg in rabbits. Echinaforce^®^, a standardized 65% alcoholic extracted from freshly harvested *Echinacea purpurea* (L.) Moench., has been reported to inhibit enveloped respiratory viruses including influenza A and B, RSV, or parainfluenza virus through neutralization with whole virions and related proteins. In the current study, Signer et al. found that four human coronaviruses were also inhibited when exposed to Echinaforce^®^, among which HCoV-229E was irreversibly inactivated at 3.2 μg/ml IC_50_ (Signer et al., 2020).

Glycyrrhizic acid, a nontoxic broad-spectrum that is derived from *Glycyrrhiza uralensis* Fisch., also provides new insights into developing anti-coronavirus therapy. In light of surface plasmon resonance (SPR) assays and NanoBit assay, disrupting the interaction the binding between the S proteins RBD and ACE2 could be a mechanism of glycyrrhizic acid (ZZY-44) to exhibit virucidal activity against SARS-CoV-2 (Yu et al., 2020). The researcher has performed a high-content screening investigation for the antiviral candidates and identified that rhizomes of *Boesenbergia rotunda* (L.) Mansf. and its bioactive compound panduratin A exert the inhibitory effect against SARS-CoV-2 infection at both pre-entry and postinfection phases (Kanjanasirirat et al., 2020). Meanwhile, treatment with this compound was able to restrain viral infectivity in human airway epithelial cells.

As we all know, *Artemisia annua* L. was an ancient Chinese herb widely applied in clinical therapeutics on account of multiple pharmacological properties, particularly in antimalarial activities. Recently, a cluster of compounds derived from this plant were revealed to exhibit inhibitory effects against SARS-CoV-2. Among nine artemisinin-related constituents, arteannuin B that acted at the post-entry step of SARS-CoV-2 showed the most prominent antiviral potential with an EC_50_ of 10.28 ± 1.12 μM. Artesunate and dihydroartemisinin, which could be clinically achieved in plasma after intravenous administration, had similar EC_50_ values of 12.98 ± 5.30 μM and 13.31 ± 1.24 μM, respectively (Cao et al., 2020). There are several antiviral ingredients in *Forsythia suspensa* (Thunb.) Vahl., of which phillyrin (KD-1) is the most representative. According to research conducted by Yang’s group, KD-1 not merely reduced replication of SARS-CoV-2 and HCoV-229E *in vitro*, but also markedly downregulated proinflammatory cytokines by the way of suppressing the NF-kB signaling pathway.

Tanshinone I, a flavonoid compound purified from the medicinal plant *Salvia miltiorrhiza* Bunge., acted as time-dependent inhibitors of PL^pro^, and furthermore exhibited the most potent nanomolar level inhibitory activity toward deubiquitinating (IC_50_ = 0.7 μM) (Park et al., 2012b). So far, the safety of tanshinone I is still under studies, whereas tanshinone IIA was observed to show severe growth inhibition, development malformation, and cardiotoxicity at high concentrations in the zebrafish normal embryos assay (Wang et al., 2017). The same author produced other studies on SARS-CoV therapeutics, compared to the former, hirsutenone isolated from *Alnus cremastogyne* Burkill. displayed good SARS-CoV PL^pro^ inhibitory activities (Park et al., 2012a). 8â-hydroxyabieta-9(11), 13 dien-12-one, and savinin demonstrated significant activity against SARS-CoVs with higher sensitivity index of above 510 from *Cornus officinalis* Siebold and Zucc. (Wen et al., 2007). The *Rheum palmatum* L. and *Glycyrrhiza uralensis* Fisch. were found to contain flavonoid emodin and glycyrrhizin, respectively, which inhibited attachment to the host cells and induced nitrousoxide synthase (Cinatl et al., 2003; Ho et al., 2007). As for their toxicity, cohort genotoxic studies have elucidated that glycyrrhizin is neither teratogenic nor mutagenic and may have properties of anti-genotoxic under the certain conditions; nonetheless, being continuously exposed to glycyrrhizin compounds at high concentration it can produce hypermineralocorticoid-like effects in both animals and humans (Isbrucker and Burdock, 2006). Another compound emodin has been proven to possess laxative effects leading to melanosis, but only at very high doses, for example, 1–3 g/kg/d for mice (Sougiannis et al., 2021). High dose of emodin can also result in mutagenic or hepatotoxicity by blocking the UGT1A1 enzyme activity (Wang et al., 2016). Kim’s group isolated two compounds against SARS-CoVs, isobavachalcone and psoralidin, displaying good SARS-CoV PL^pro^ inhibitory activities (Kim et al., 2014). Psoralidin is a nontoxic but low oral bioavailability compound (Sharifi-Rad et al., 2020).

These seem to be promising compounds from some new research on MERS-CoVs. Lin et al. (2017) found that N protein that is necessary for MERS-CoVs replication was decreased after resveratrol therapeutics. Furthermore, resveratrol could downregulate the apoptosis caused by MERS-CoVs *in vitro*. Lycorine and Emetine also showed the high activity against MERS-CoVs with low IC_50_ of 1.63 and 0.34 μM, respectively (Shen et al., 2019).

## Advanced Strategies on Screening Extracts Against CAPs

High-throughput screening (HTS) is a fragment-based screening product of multidisciplinary integration, which is one of the most active techniques in areas of medical science in recent years. It ties merits of efficient, generally applicable, automatic, and high specificity in a particularly computational way that is superior to the conventional method and offers a new powerful tool for candidate drugs broad-spectrum screening. There are two key features to handle compounds with HTS: miniaturization and automation. In miniaturization, it has a standard format plate and each of these wells is another experiment which even can do 1,536 experiments on a single plate. Then, HTS executes the process on the microplate with automated operating systems and analyzes and processes the experimental data via detection instrument and software algorithm. Commonly, scintillation proximity assay and fluorescence assay are widely used in screening. A quenched fluorescence resonance energy transfer assay was developed to evaluate the activity of 3CL^pro^ in the presence of 50,000 small drug-like molecules on a fully automated system. In secondary studies, it remained five novel molecules that exhibited inhibitory activity (IC_50_ = 0.5–7.0 μM) toward 3CL^pro^ through a series of virtual and experimental filters (Blanchard et al., 2004).

The screening model is also critical for HTS, especially in recognizing the interaction between drugs and molecular targets and the basic mechanism of drug. At present, these models mainly focus on receptors, channels, and various cellular responses. The majority of the HTS virology assays follow a standard paradigm, which are cell-based, phenotypic screens designed to identify antiviral compounds with a broad range of mechanisms (Wen et al., 2019; Chojnacka et al., 2020). Wen et al. (2019) have succeeded in establishing an automated plaque reduction neutralization assay to determine neutralization titers of anti-RSV antibodies that allow simultaneous titration of a large number of samples in a shorter time. This higher throughput automatic counting method proved to produce more precise and reliable titers than current methods, which greatly benefit drug/vaccine candidate screening.

A cell-based HTS assay was reported to search for inhibitors of IFV. In this study, the authors set up 293T cell lines that constitutively synthesize negative strand RNA, which expresses Gaussia luciferase upon IVA infection, for which 2000 small molecules screening and 17 compounds exhibited 90–100% inhibition of luminescence signal for a rate of 0.85% (Gao et al., 2014). Another similar study used the HTS platform based on vRNA promoter luciferase reporter plasmid to identify three medicinal plants that could significantly inhibit promoter transcription activity due to the procyanidin (Dai et al., 2012).

### Virtual Screening

Virtual screening encompasses all sorts of computational techniques that allow cutting a huge virtual library to a more manageable size (Walters et al., 1998). Nowadays, a large scale of algorithms offer strategies and show their unique advantages to work out modern structure-based drug designing problems because of the diversity in both their accuracy and computational speed.

Molecular docking is one of the most extensively used computational approaches, whereby large virtual libraries of chemical compounds are shrunk in size to a manageable subset, and place of the putative “ligands” into the appropriate site that creation of a negative image of the target site, ultimately ‘score’ their potential complementarity to binding sites (Kuntz, 1992). Characterization of the binding behavior plays a significant role in rational design of drugs and in the elucidation of the fundamental biochemical process. Nevertheless, there still remain some awkward issues urgently needed to be addressed, especially with regard to current scoring schemes. At present, nine docking programs, namely, AutoDock, Flex, Fred, Glide, Gold, Slide, Surflex, and QXP, have been widely tested to evaluate their potency in drug discovery applications (Kellenberger et al., 2004; Kitchen et al., 2004).

Some cases of screening potential antiviral compounds with molecular docking have been summarized in Table 4.

### Network Pharmacology

As bioinformatics moves far ahead, systems biology and pharmacology can be thought of as a promising network-based approach toward more effective drug development (Jia, et al., 2009; Schadt et al., 2009). Network pharmacology highlights a paradigm shift from the current “one target, one drug” strategy to a novel version of the “network target, multi-components” strategy (Li and Zhang, 2013). Network-based drug discovery is regarded as a potential method toward more cost-effective drug development with the rapid progress. From the perspective of systems biology, system pharmacology can map the “disease-target-drug” to the network level, and screen for the lead compound through further calculation, analysis, and modeling, observe the intervention and influence of drugs on the network.

The introduction of “networks” in drug discovery, including assessments of network topology and dynamics, provides a quantifiable description of complicate systems and its response to a variety of herbal treatments. One of the greatest strengths of pharmacological network is that facilitating the discovery of new drugs mechanism from static and dynamic aspects respectively. A visual toolbox has provided the interaction relationship that integrates various information, including drugs, genes, targets, diseases, and other information in an abstract way (Hopkins, 2008). In the network composed of multiple levels, the element components shape into nodes, and the interaction forms into the connection between nodes. Common tools widely used to analyze the necessary information include in the field of TCMID research, Cytoscape, GUESS, Pajek, and VisANT (Xue, et al., 2013; Zhou et al., 2020b). As far as research on effective drugs targeting coronavirus, Zhou et al. presented a study that prioritized 16 potential anti-SARS-CoV/SARS-CoV-2 repurposable drugs that are further validated by enrichment analyses of drug–gene signatures and CoV-induced transcriptomics data in human cell lines based on a poly-pharmacology network platform that quantifies the outcomes between the virus–host interaction and drug targets in the PPI network (Zhou et al., 2020a).

Given the crisis in commercial translation, network pharmacology offers a new framework on how to innovate drug discovery, and thus it is an idea whose time has come, yet such strategies are at present a minority activity in the pharmaceutical industry.

## Conclusion and Future Prospects

Large cohort studies have provided direct evidence that viruses represent a common cause of CAP and three types of viruses IFVs, RSVs, and CoVs should be the most responsible for this. Vaccination is now the primary strategy for virus epidemic. Nonetheless, drug therapy is still the critical approach for CAPs in patients on account of antigenic shift. Few drugs are now approved by FDA administered during this viral infection and symptoms remission from severe CAPs. RSV infections in high-risk young children and newborns have been prevented successfully with palivizumab and Ribavirin. For IFVs, two classes of agents are internationally accredited agents for treatment, namely the adamantanes and neuraminidase inhibitors. However, the former lost their potency over time due to the rapid occurrence of drug resistances. Until now, there are no miracle drugs as therapeutic for HCoVs. The characteristics on novel coronavirus are as follows: 1) it becomes more transmissible than SARS-CoV; 2) the pathogenicity is heavier than influenza, but lighter than SARS; and 3) the detoxification time is longer. Hence, it is urgently necessary to develop target drugs to lead the response to a global public health emergency result from frequently mutational viruses like the case of SARS-CoV-2.

Naturally based pharmacotherapy plays a profound role in the treatment of viral pneumonia and, indeed, most of plant secondary metabolites and their derivatives also have a desired effect on antivirus activity. This overview used SciFinder^®^ and PubMed to search for any article published between 2003 and 2020 that is relevant to plant-derived natural products for the prevention and treatment of viral pneumonia, in particular those caused by three types of virus that we focus on. A total of 62 types of compounds were summarized and classified in the tables. Of note, quercetins and flavonoids are phytochemicals of plant origin which have known antiviral properties to diminish the replication of many viruses like IFV and RSV.

While plaque assays are the standard tools to measure infectious virus, the methodology is time-consuming and requires experience in recognizing plaques (Wen et al., 2019). The assays are also prone to variation among analysts due to plaque recognition and manual counting errors. Here, we introduce three different advanced methods and offer a new mentality on screening antiviral drug with high effect and low toxicity. Based on HTS technology, a pharmacologically quantitative analysis is used in tandem with the established cell model to identify compounds that bind to target proteins and are thus potential new drugs. Another virtual screening method has provided a tool to enhance basic scientific research that promoted drug discovery projects, resulting in marketed pharmaceutical products. Through molecular docking, a series of small molecules, including natural compounds, have been screened and confirmed to directly inhibit these important proteins in SARS or MERS coronavirus. Network pharmacology analysis is generally followed by molecular docking. This approach can provide information with drug–protein interaction.

Owing to the side effects of synthetic medicine, researchers turn to herbal remedies for accessible and economical treatment of viral diseases, which comparatively bear fewer chances of toxicity and resistance.
