# Supplementary material for: Overview of Viral Pneumonia Associated With Influenza Virus, Respiratory Syncytial Virus, and Coronavirus, and Therapeutics Based on Natural Products of Medicinal Plants
Source: Front Pharmacol. 2021 Jun 21;12:630834. doi: 10.3389/fphar.2021.630834 (PMC8256264; doi:10.3389/fphar.2021.630834)
Supplement: Supplementary file 1 [file Image2.pdf]

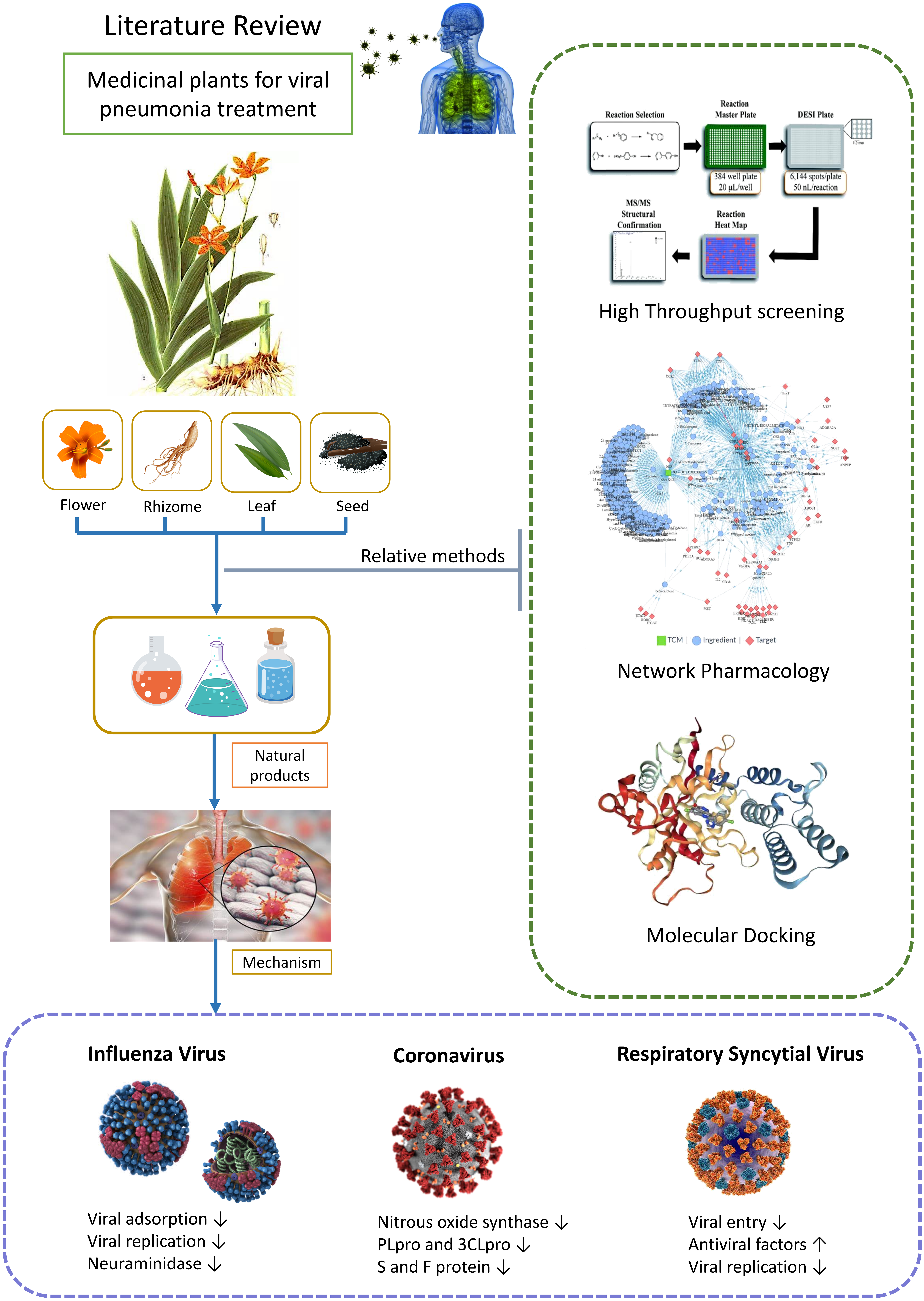

Literature Review
Medicinal plants for viral pneumonia treatment
Flower
Rhizome
Leaf
Seed
Relative methods
Natural products
Mechanism
Reaction Selection
Reaction Master Plate
DESI Plate
384 well plate 20 µL/well
6,144 spots/plate 50 nL/reaction
MS/MS Structural Confirmation
Reaction Heat Map
High Throughput screening
TCM | Ingredient | Target
Network Pharmacology
Molecular Docking
Influenza Virus
Viral adsorption ↓
Viral replication ↓
Neuraminidase ↓
Coronavirus
Nitrous oxide synthase ↓
PLpro and 3CLpro ↓
S and F protein ↓
Respiratory Syncytial Virus
Viral entry ↓
Antiviral factors ↑
Viral replication ↓
