# Supplementary material for: Overview of Viral Pneumonia Associated With Influenza Virus, Respiratory Syncytial Virus, and Coronavirus, and Therapeutics Based on Natural Products of Medicinal Plants
Source: Front Pharmacol. 2021 Jun 21;12:630834. doi: 10.3389/fphar.2021.630834 (PMC8256264; doi:10.3389/fphar.2021.630834)
Supplement: Supplementary file 2 [file Image1.pdf]

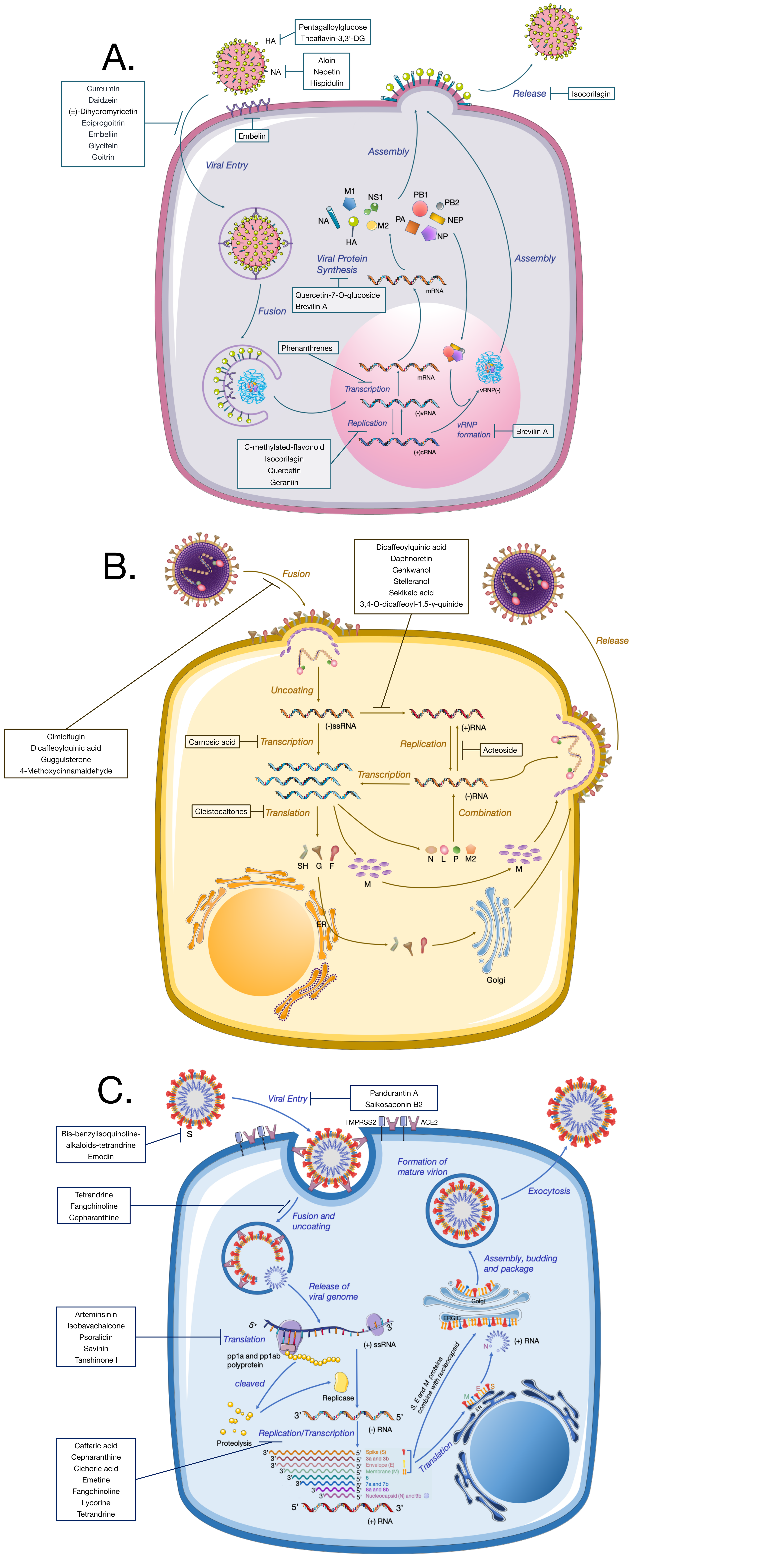

FIGURE 1 | Replication Mechanisms of Three Different Kinds of Virions. A diagram of the viral lifecycle of (A) influenza virus, (B) respiratory syncytial virus, and (C) coronavirus, indicating where each therapeutic exerts antiviral activity. The therapeutic targets of natural products derived from medicinal plants are shown in bold.
